# Marine Oil Slick Detection Based on Multi-Polarimetric Features Matching Method Using Polarimetric Synthetic Aperture Radar Data

**DOI:** 10.3390/s19235176

**Published:** 2019-11-26

**Authors:** Guannan Li, Ying Li, Bingxin Liu, Peng Wu, Chen Chen

**Affiliations:** 1 Navigation College, Dalian Maritime University, Dalian 116026, China; lgn@dlmu.edu.cn (G.L.); gisbingxin@dlmu.edu.cn (B.L.); 18840866641@163.com (P.W.); chenchen_dlmu@126.com (C.C.); 2 Environmental Information Institute, Dalian Maritime University, Dalian 116026, China

**Keywords:** oil spill detection, polarimetric synthetic aperture radar, multi-polarimetric features model, spectral pan-similarity measure

## Abstract

Polarimetric synthetic aperture radar is an important tool in the effective detection of marine oil spills. In this study, two cases of Radarsat-2 Fine mode quad-polarimetric synthetic aperture radar datasets are exploited to detect a well-known oil seep area that collected over the Gulf of Mexico using the same research area, sensor, and time. A novel oil spill detection scheme based on a multi-polarimetric features model matching method using spectral pan-similarity measure (SPM) is proposed. A multi-polarimetric features curve is generated based on optimal polarimetric features selected using Jeffreys–Matusita distance considering its ability to discriminate between thick and thin oil slicks and seawater. The SPM is used to search for and match homogeneous unlabeled pixels and assign them to a class with the highest similarity to their spectral vector size, spectral curve shape, and spectral information content. The superiority of the SPM for oil spill detection compared to traditional spectral similarity measures is demonstrated for the first time based on accuracy assessments and computational complexity analysis by comparing with four traditional spectral similarity measures, random forest (RF), support vector machine (SVM), and decision tree (DT). Experiment results indicate that the proposed method has better oil spill detection capability, with a higher average accuracy and kappa coefficient (1.5–7.9% and 1–25% higher, respectively) than the four traditional spectral similarity measures under the same computational complexity operations. Furthermore, in most cases, the proposed method produces valuable and acceptable results that are better than the RF, SVM, and DT in terms of accuracy and computational complexity.

## 1. Introduction

The oceans play an important role in the global ecosystem, as they affect the global ecological balance and provide resources and energy. Pollution of the ocean surface by mineral or petroleum oil is a major environmental problem [1,2]. The main causes of marine floating oil slicks can be divided into two categories. The first category includes oil leakage as a result of human activity such as shipping accidents, local leakage near ports, and oil well explosions. The second includes fairly slow and relatively constant natural seepage of oil from the seabed [2,3,4]. As much as half the oil that enters the coastal environments come from natural oil and gas seeps. Furthermore, natural oil seeps are by far the single largest source of oil in the marine environment, accounting for approximately 47% of the total annual release of petroleum compounds [3,5,6]; they are also the only natural source of oil entering the environment. The ability to detect and track oil slicks floating on the ocean surface has attracted the attention of researchers due to their effects on, for example, marine ecosystems, wildlife, and aquaculture. [6,7].

Remote sensing technology can be used effectively for widespread and rapid ocean surveillance. Compared with conventional ground-based monitoring techniques, remote sensing is an effective way to monitor, track, and map targets at a regional scale due to its wide synchronous coverage and cost effectiveness [8]. As a tool that can be used at all times of day and in all weather conditions, polarimetric synthetic aperture radar (Pol-SAR) can provide valuable information using its own active illumination systems and multiple imaging and polarization modes [2,9,10,11]. This increases the range of applications for Pol-SAR data and means that effective information can be retrieved from targets with different characteristics and for various research demands. Pol-SAR systems can measure the scattering matrix and obtain sufficient intensity and coherent phase information for each resolved image pixel because the electromagnetic pulse signals are transmitted in two orthogonally polarized fields [7]. Therefore, the quad-polarization SAR system retains all the scattering information data that describes the polarization characteristics of each resolved image pixel based on the vector characteristics of the scattered field. However, despite the fact that it can be used to obtain abundant and comprehensive target information, it is a challenge for quad-polarization SAR systems to be comprehensive considering the balance of antenna technology, system power consumption, size of the processed swath, data download, and processing efficiency due to the doubled pulse repetition frequency [12,13,14]. Despite this, Pol-SAR is widely used to monitor the marine environment.

Many studies have explored oil spill mapping and monitoring while focusing on different oil spill accidents, data sources, and algorithms. Changes in polarization over an oil slick can be used to describe the characteristics of an oil slick while multi-feature analysis and classification can improve classification accuracy. Skrunes et al. [15] compared and analyzed eight well-known multi-polarimetric features of SAR data for a mineral oil spill versus a biogenic slick at the Frigg field situated northwest of Stavanger, which provided reference for feature analysis and selection in oil spill detection. Migliaccio et al. [16] summarized quad-polarimetric features describing expected behaviors over slick-free, weak-damping slick-covered, and oil-covered ocean surfaces, verified the effectiveness of these polarimetric features, and also provided reference for the application and analysis in oil spill detection for this paper. Latini et al. [17] quantitatively compared and analyzed the Deepwater horizon oil spill accident in the Gulf of Mexico based on different polarimetric SAR systems and evaluated the oil spill characteristics of low-noise systems. That work was a forward-looking study of the effects of different bands and noise on oil spill detection. Li et al. [18] proposed an oil spill detection architecture based on the staked auto encoder, evaluated the performance of different filters, and pointed out the need to study the information of data from combined co-polarization channels due to the low signal-to-noise ratio of the cross-channels. Buono et al. [19] comprehensively analyzed the influence of incident angle, wind speed, and noise equivalent sigma zero (NESZ) on the sensitivity of co-polarized channel parameters, which laid the foundation for the study of oil spill detection under different SAR acquisition and surface parameters. For natural oil seepage detection, Pellon de Miranda et al. [20] presented results obtained using an unsupervised semivariogram textural classifier to detect leaks in the Gulf of Mexico based on the Radarsat-1 data sets. Suresh et al. [21] proposed a framework for detecting natural oil seepages and estimating their source, thereby contributing to the comprehensive analysis of natural oil spills in the future. Nunziata et al. [22] comprehensively evaluated the effects of SAR imaging parameters and environmental conditions on natural oil seep in Mexico using single-polarization SAR images, providing the valuable experimental conclusions for the comprehensive study of natural oil seepage. Some studies have analyzed oil spill mapping and scattering characteristics based on the same data used in this work and interpreted the visible dark spots in the SAR image as natural crude oil seeps. Zhang et al. [7] improved the parameters used to estimate soil moisture via compact polarization data and proposed a simple and effective detection technique based on a natural oil seeps detection parameter suitable for full polarization data. Li et al. [23] exploited compact Pol-SAR to monitor oil slicks at sea, analyzed the compact Polarimetric scattering mechanisms of oil seeps and Deep-Water Horizon oil spill, and pointed out that the natural oil seeps exhibited a change in scattering mechanisms from that of the Deep-Water Horizon oil spill. Buono et al. [24] analyzed the scattering characteristics of natural oil seeps under different SAR architectures (full-polarimetric, hybrid polarity, and π/4 modes) and evaluated their performance relative to full polarization; Guo et al. [25] proposed a CNN method to identify oil slicks and look-alikes based on polarimetric features and reached higher detection rates than traditional methods, which is effective for detecting and distinguishing natural oil seeps.

However, these studies paid more attention to polarimetric feature extraction and used them as independent inputs for machine learning rather than for screening the optimal contribution features by comprehensively assessing the ability of the polarimetric features most frequently employed to discriminate between oil slicks and seawater. Mindful of this limitation, some researchers have taken new perspectives inspired from spectral similarity measures to obtaining the target information curve for classification, and they have achieved good results using different polarizations of multi-temporal SAR [8,26]. The spectral similarity measures are initially applied to hyperspectral image processing, which includes two main categories: uncertainty measures (includes spectral angle and spectral distance) and randomness measures (includes spectral information divergence and correlation coefficient measure) [27,28,29,30]. The concept and definition of spectral similarity measures is that the homogeneous unlabeled pixels are searched for, matched, and assigned to a class with the highest similarity to their spectral information [26,27,28]. Many studies have demonstrated the effectiveness of spectral similarity measures in oil spill detection based on hyperspectral images [27,31,32]. Yang and Xu applied the spectral similarity measure to multi-temporal SAR images to match targets with similar backscattering intensity curve and obtain the crop classification results [8,26]. These methods apply the spectral similarity concept to target classification on SAR image and obtain valuable and good results, which are a referenced and forward-looking study for target detection on SAR system. However, these methods pay more attention to the backscattering intensity. In oil spill detection, the polarimetric SAR system can provide obtained abundant and comprehensive target information, which is also the focus of this paper. Thus, in this paper, we propose making a further step to extend the spectral similarity concept to polarization information.

To summarize, on the one hand, the application of spectral similarity measure is still lacking in polarimetric SAR data. On the other hand, comparisons and analysis between thick and thin oil slicks are scarce. In addition, most methods of these studies are applied to oil spill images obtained from different sensors, different times, and different sea areas. The difference of SAR sensors designs and detection conditions may affect the effectiveness and classification accuracy of the method [2,33]. Here, brief notes and inspiration from the work in spectral similarity measure based on SAR images [8,26], as well as an improvement and enhancement to forms the background to the present study. It should be emphasized that the two oil spill images used in this paper were taken from the same scene of the Radarsat-2 image. This was done to verify the effectiveness of the proposed method using the same sensor, time, and research area. This is a large step in preventing errors caused by different image and detection conditions. These two things contributed in two aspects. First, the typical polarimetric features were summarized and selected based on the Jeffreys–Matusita (J–M) distance to obtain optimal feature sets by comprehensively assessing the ability to distinguish between thick and thin oil slicks and seawater. Furthermore, the multi-polarimetric features curves of these targets (thick oil slick, thin oil slick, and seawater) were generated based on the optimized polarimetric features set. Second, a multi-polarimetric features model matching method based on spectral pan-similarity measure (SPM) was proposed that could identify oil spills and seawater based on their polarimetric feature curves. The spectral measure matching method has not been referenced or tested from this perspective in any previous oil spill identification research. Furthermore, the SPM combines spectral vector size, spectral curve shape, and spectral information content, which can obtain classification results of a higher accuracy than traditional single spectral similarity measures because it is based on more comprehensive similarity information. Therefore, this study proves the superiority of the spectral measurement method based on multi-polarimetric features for oil spill detection research for the first time after comprehensively evaluating the results of several other spectral measurement methods.

## 2. Experimental Dataset and Study Sites

### 2.1. Dataset Overview

**Figure 1:**
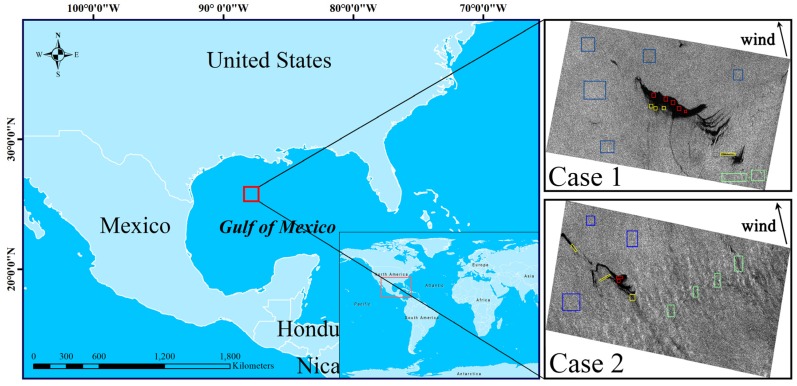
Location of the oil spill. Images taken using the quad-polarization Radarsat-2. The colored boxes indicate sample regions used in statistical analysis and modeling (blue: sea, red: thick oil region, yellow: thin oil region, green: look alike).

RADARSAT-2 provides many operation modes and polarizations, including, e.g., Spotlight, Fine, Wide, Standard, and ScanSAR, in linear single-, dual-, and quad-polarizations. The quad-polarization RADARSAT-2 data, which incorporates extremely low background noise and cross-talk correction for different channels, provides coherent phase information and intensity for all the channels (VV, VH, HV, and HH) [7,23]. We used two separate parts of a C-band single look complex (SLC) fine quad-polarization RADARSAT-2 image acquired over well-known oil slicks in the Gulf of Mexico at 12:01 UTC on May 8, 2010. The visible dark areas relate to the oil slicks, which were interpreted as natural oil seeps that occur frequently in this area, as shown in Figure 1. The wind speed was 6.5 m/s, which is optimal condition for detecting oil slicks via SAR imaging [7,34], and the wind direction was 167° (approximately south wind), as observed by and obtained from buoy #42047 (27°53′48″ N 93° 35′50″ W) of the National Oceanic and Atmospheric Administration’s National Data Buoy Center [7,23]. The SAR data used in this study are further characterized in Table 1.

### 2.2. Sample Data Acquisition

Some studies based on experiments and analysis have shown that the damping ratio of mineral oil increases as the thickness of an oil slick layer increases, and that a thick oil slick has a lower backscattering intensity than a thin oil slick. Moreover, under action from wind, an oil slick on the leeward side is much thicker than that on the windward side; therefore, there is a visible dark line on the leeward side edge of an oil slick while a “feathered” oil slick is usually produced on the windward side [35,36,37]. In addition, other physical phenomena can also generate a weak-damping low-backscattering area, known as a “look-alike”, which is the primary cause of false alarms for oil slick extractions. Such phenomena include low wind areas (large dark areas with a fuzzy boundary) and internal waves (which appear as bright-dark strips) [38,39]. As noted earlier, the wind direction at the time were taken for this study was approximately south wind; therefore, the northern region on the leeward side, which had an obvious dark boundary, was selected as the thick oil slick; the feathered tail areas of the southern windward side and the oil slick strips were selected as thin oil slicks; and the bright-dark strips caused by ocean waves were selected as look-alikes, as shown in Figure 1.

In this study, we took the images from Case 1 for modeling and precision evaluation, for example; we randomly selected 10,000 sample points from the thick and thin oil regions and clean seawater, respectively, using the “Create Random Points” tool in ArcGIS 10.2; we also took 2000 sample points from the look-alike area. Of these, 50% of the data was utilized for statistical analysis and modeling, while the other 50% was utilized for accuracy verification, thus ensuring the independence of the training and testing samples. In the calculations to obtain the polarimetric features for study, multi-looked (3 × 3 window) and a Boxcar filter (3 × 3 window) were utilized to process the Pol-SAR images based on the results of window size analysis in the literatures [25,40].

## 3. Methodology

**Figure 2:**
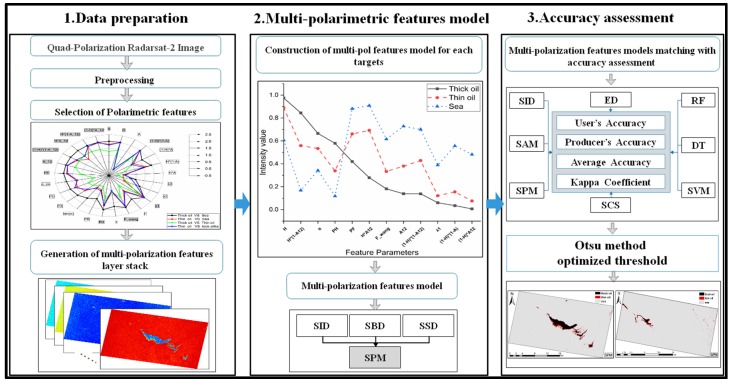
Schematic of the overall workflow.

A flow chart showing the proposed new oil spill classification method using Radarsat-2 images is given in Figure 2. It is divided into three main parts: data preparation, multi-polarimetric features model generation and matching, and accuracy assessment and result acquisition.

In the first part, the optimized multi-polarimetric features are extracted. This includes radiometric calibration, geocoding, polarimetric filtering, polarimetric decomposition, polarimetric feature extraction, and features selection. The multi-polarimetric feature layer stack is then used as the input variable for further processing.

In the second part, the multi-polarimetric features model is generated by considering the intensity curve of the multi-polarimetric features of the target(s) (oil slicks and seawater). The spectral similarity measure can be utilized to evaluate the spectral difference between the known reference target and unknown target. However, the theoretical analyses and experimental results of some studies have shown that spectral similarity cannot be adequately characterized by a single index of spectral similarity [27,28]. Hence, in this part of this study, we propose the spectral pan-similarity measure (SPM) matching model based on multi-polarimetric features. The concept, construction, and definition of SPM is that two spectral curves are considered to be similar when satisfying the three conditions with similar spectral vector size, similar spectral curve shape, and similar spectral information content [27,28]. This is used to find the intrinsic polarimetric feature curve pattern for each target class as this integrates the magnitude of the spectral vector, curve shape, and information content, which provides more comprehensive information than other spectral similarity measurements [8,26,27,28].

In the third part, an accuracy assessment is performed by comparing the results from the proposed method with those from other spectral matching measures and classifiers using the images from Case 1 and 2. Finally, the multi-polarimetric features matching model is used to obtain the final classification results.

### 3.1. Extraction and Selection of Polarimetric Features for Marine Oil Spill Detection

The interpretations and analysis of polarization information can capture important indicators of scattered signals from the oil slicks [7,11,23,34,41]. Many prior studies have demonstrated the potential and advantages of polarization decomposition using a polarimetric SAR scattering matrix to analyze the scattering properties of an oil slick. The scattering matrix *S* is given as follows [11,34,41]:

(1) $$S=\left[\begin{array}{cc} S_{HH} & S_{HV}\\ S_{VH} & S_{VV} \end{array}\right]$$

Here, the elements *S_ij_*, with *i*, *j* ∈ {*H*,*V*}, where *i* represents transmit, *j* represents receive, and H and V represent horizontal and vertical polarizations, respectively [2,34]. In the case of backscattering from a reciprocal medium, the relationship *S_HV_* = *S_VH_* is satisfied. The three-dimensional Pauli-basis vector *k* contains the same information as the scattering matrix, which can relate the polarimetric backscatter information to the physical properties of the scattering target [11,17]. In this study, the polarimetric dimension is three. The averaged coherence matrix *T*_3_ can be constructed based on the outer product of the Pauli scattering vector *k* with its conjugate transpose k^*T^, which can be obtained from the scattering matrix *S*. The vector *k* and matrix *T*_3_ are given by [41,42]:

(2) $$k=\frac{1}{\sqrt{2}}{\left[S_{HH}+S_{VV},S_{HH}-S_{VV},2S_{HV}\right]}^{T}$$

(3) $$T_{3}=\langle k\cdot k^{*T}\rangle$$

Furthermore,

(4) $$\langle T_{3}\rangle =\langle k\cdot k^{*T}\rangle =\left[\begin{array}{ccc} T_{11} & T_{12} & T_{13}\\ T_{21} & T_{22} & T_{23}\\ T_{31} & T_{32} & T_{33} \end{array}\right]\;=\frac{1}{2}[\begin{array}{ccc} \langle {\left|S_{HH}+S_{VV}\right|}^{2}\rangle & \langle \left(S_{HH}+S_{VV}\right){\left(S_{HH}-S_{VV}\right)}^{*}\rangle & 2\langle S_{HH}+S_{VV}\rangle S_{HV}^{*}\\ \langle \left(S_{HH}-S_{VV}\right){\left(S_{HH}+S_{VV}\right)}^{*}\rangle & {\langle \left|S_{HH}-S_{VV}\right|}^{2}\rangle & 2\langle S_{HH}-S_{VV}\rangle S_{HV}^{*}\\ 2\langle S_{HV}{\left(S_{HH}+S_{VV}\right)}^{*}\rangle & 2\langle S_{HV}{\left(S_{HH}-S_{VV}\right)}^{*}\rangle & 4\langle {\left|S_{HV}\right|}^{2}\rangle \end{array}]$$

where *N* represents the number of samples included in the average and <⋅> denotes the ensemble average.

A diagonalized form of the coherence matrix can be obtained from the eigenvector (eigenvalue), computed from the Hermite averaged coherence matrix *T*_3_, which represents the statistical independence between the set of vectors. The average coherence matrix *T*_3_ can be further expanded into the sum of three independent objects, which are respectively described by the corresponding scattering matrices. The decomposition process for the average coherence matrix is given as follows [41]:

(5) $$T_{3}=U_{3}\sum U_{3}^{-1}=\overset{i=3}{\underset{i=1}{\sum }}\lambda_{i}T_{3i}=\overset{i=3}{\underset{i=1}{\sum }}\lambda_{i}u_{i}u_{i}^{T^{*}}$$

where ∑ is the three-dimensional diagonal matrix, u*_i_* are the orthogonal unit eigenvectors, and *λ_i_* are the eigenvalues of the coherent matrix.

The polarimetric features can be obtained using polarimetric matrices. Previous studies have shown that polarimetric features can help distinguish between oil slicks and ambient seawater. The polarimetric features employed in this study, and their definitions, expected behavior over the sea with and without oil slicks, and references are listed in Table 2.

It is not necessary to use every Pol-SAR feature in the target recognition and classification process because every feature varies in its ability to distinguish between oil slicks and seawater, and even between thick and thin slicks. The J–M distance is an index used widely to measure similarities in the field of pattern recognition and oil slick detection based on an SAR image, which is simple and has good universality [43,44,45,46,47]. The advantage of the JM distance is the fact that it is a simple and easily implemented criterion, which have a fixed range of values between 0 and 2 [45]. The exponential factor in JM distance definition gives an exponentially decreasing weight to increasing separation between the classes [48], then the JM distance will have a saturation behavior with the increase of the degree of separation due to the contribution of the exponential character, which overcomes the limitation of the transformation divergence [48,49]. The JM distance has been demonstrated to be effective, easily implemented, and good universality in polarimetric features selection for oil spill detection [44,46,47].

In this study, the J–M distance is chosen as the separability measure method on the basis of their ability to distinguish between thick slicks, thin slicks, and seawater. The implementation of JM separability measure assumes that the data distributions involved are multivariate normal distribution [45,48,49]. In the case of multivariate normal distribution, the J–M distance is defined as follows [45,48,49]:

(6) $$J_{ij}=2\times \left(1-\exp\left(-D_{ij}\right)\right)$$

where

(7) $$D_{ij}=\frac{1}{8}{\left(m_{i}-m_{j}\right)}^{T}{\left[\frac{\sum_{i}+\sum_{j}}{2}\right]}^{-1}(m_{i}-m_{j})+\frac{1}{2}\ln\frac{\left|\frac{\sum_{i}+\sum_{j}}{2}\right|}{\sqrt{\left|\sum_{i}\right|\left|\sum_{j}\right|}}$$

Here, *J* represents the J–M distance for the feature in this study; *m_i_* and *m_j_* represent the mean vector of a certain feature value for different types of selected targets training samples; and *∑_i_* and *∑_j_* represents the covariance matrices of the feature value for two kinds of different ground targets training samples. The value of the J–M distance ranges from 0–2. When the J–M distance is high, the separability between different ground targets is greater and vice versa. When the J–M distance is greater than 1.9, the two ground targets have strong separability; values ranging from 1–1.9 represent good separability, and those ranging from 0–1 represent weak separability [46].

**Figure 3:**
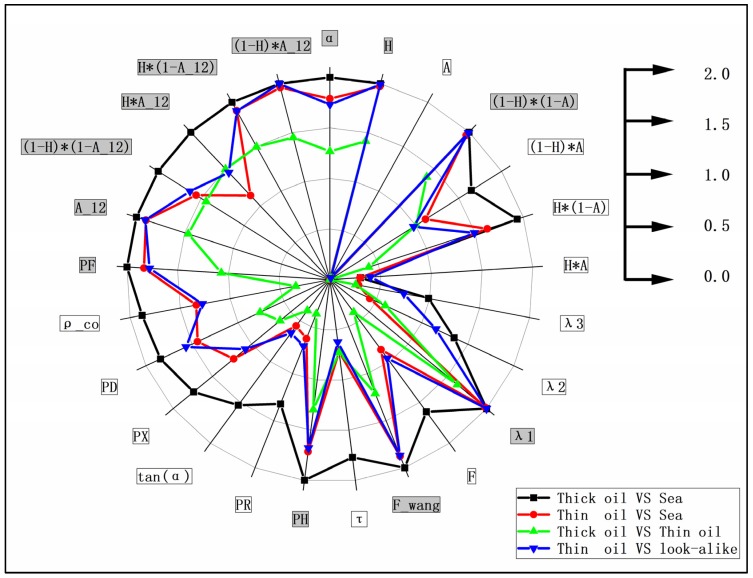
Jeffreys–Matusita distance for different polarimetric features in Case 1.

**Figure 4:**
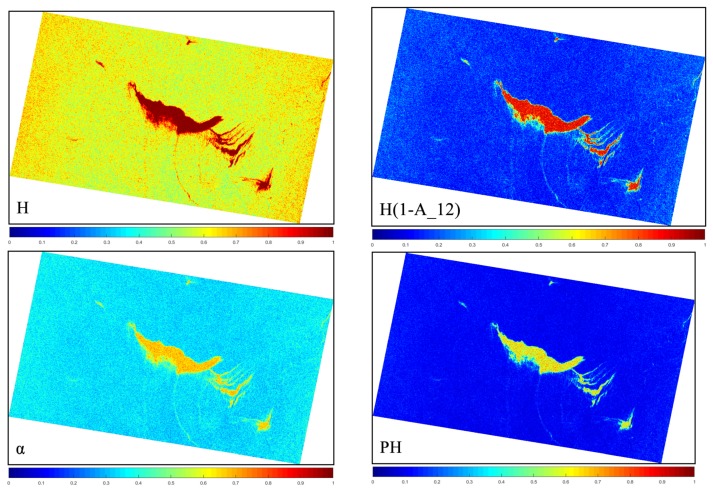

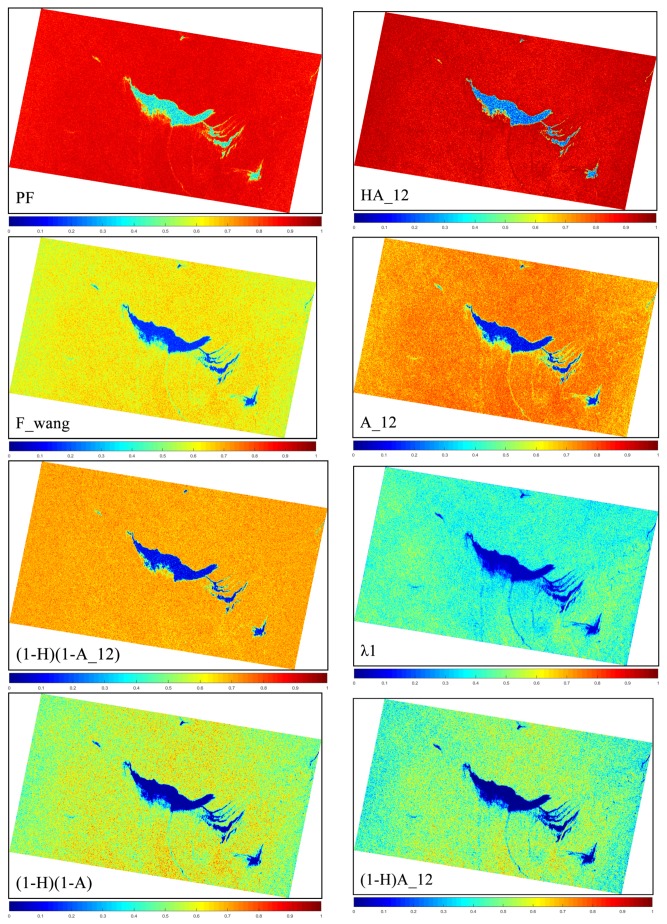
Multi-polarimetric features selected by the results of the Jeffreys–Matusita distance for different targets.

In this study, as described in Section 2.2, training samples for different targets were screened from the ROIs of corresponding targets using the “Create Random Points” tool in ArcGIS 10.2, in order to avoid the impact of the specificity of the data in a specific ROI on the statistical analysis of the data. The J–M distance between different targets samples in Case 1 of the Radarsat-2 image was calculated to evaluate the capability of the polarimetric features listed in Table 2. The J–M distances for the different regions and 25 polarimetric features in Case 1 of the Radarsat-2 image are shown in Figure 3. The difference between the J–M distances of the different parameters and different targets is significant; the difference between thick and thin oil, in particular, is generally low, approximately 0.01–1.48. The best results for the J–M distance exceeded 1.8. As described above, the J-M distances exceeded 1 represents good separability between two targets. Therefore, the set of polarimetric features with a J–M distance value greater than 1 were selected for subsequent modeling and analysis in Figure 3 (marked with a gray background), which represents good separability between both thick oil slicks vs. seawater, thin oil slicks vs. seawater, thick oil slicks vs. thin oil slicks, and thin oil slicks vs. oil spill look-alikes. It should be noted that the J–M distance value for screening polarimetric features may vary under different conditions, which depended on the differences between targets. Figure 4 presents the result for the selected polarimetric feature set. For a logical comparison, all of the selected features are normalized to 0–1.

### 3.2. Multi-Polarimetric Feature Model of Oil Slick Identification

This study used the spectral similarity measures and multi-polarimetric features models based on Radarsat-2 image to identify oil slicks. The proposed method extracts the multi-polarimetric feature intensity vector from the dataset selected in the previous section and treats the feature intensity vector as a feature curve. This algorithm defines the similarity measure between a known reference pixel and an unknown target pixel. If the feature curve of the unlabeled pixel is very similar to that of the multi-polarimetric features model, then the pixel is assigned to the class to which the latter belongs. The brief description of other work on spectral similarity matching, provided in Section 1, is the inspiration for and forms the background of this study.

**Figure 5:**
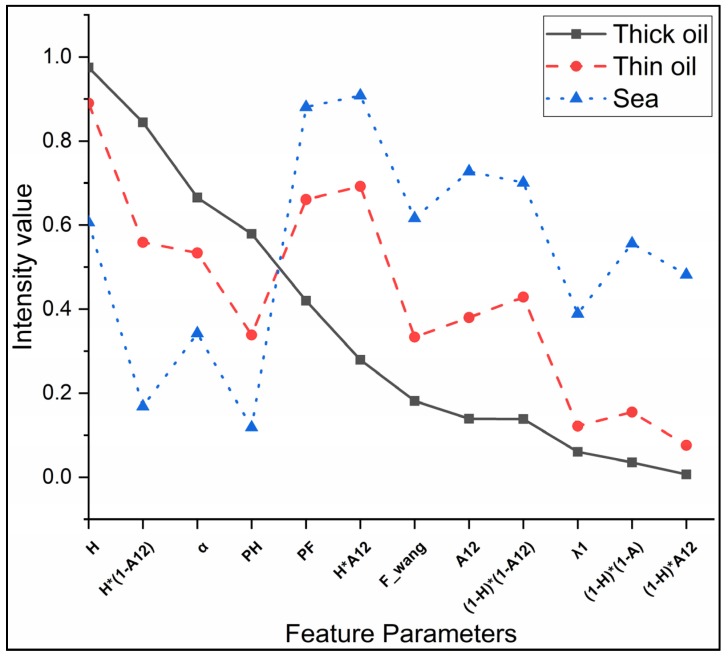
Polarimetric feature center intensity curve based on the selected parameters for three target types.

Oil slicks and seawater exhibit different characteristics under different polarimetric features due to differences in their intrinsic scattering mechanisms. The curve for targets under the multi-polarimetric feature set selected based on the J–M distance presents its own trend characteristics. However, previous studies have concluded that, in reality, the polarimetric characteristics performance of an oil slick are affected by factors including SAR system acquisition parameters (e.g., incident angles, NESZ, and resolution), ocean environmental conditions (e.g., wind speed/direction, sea currents, waves, sea temperature, and seawater composition), and intrinsic oil slick information (e.g., thickness, oil type, weathering degree, and cause of formation) [2,15,19,29,59,60,61]. Therefore, considering the situation described above, the proposed method uses statistical analysis to select the mean of the target sample region in order to construct the multi-polarimetric features model. The multi-polarimetric features models of the image in Case 1 can be represented graphically. For a logical comparison, all of the selected features are normalized to 0–1, as shown in Figure 5.

The spectral pan-similarity measure (SPM) combines the magnitude, shape, and information about the polarimetric feature vector including the vector distance, vector correlation coefficient, and relative entropy [28]. Suppose *x^i^* = (*x^i^*_1_, *x^i^*_2_, *x^i^*_3_, …, *x^i^_N_*)^T^ represent the known target (oil slick) polarimetric feature vector curve in the image, and *x^j^* = (*x^j^*_1_, *x^j^*_2_, *x^j^*_3_, …, *x^j^_N_*)^T^ represent the unknown target polarimetric feature vector curve extracted from the image, where *N* is the band dimensionality of the polarimetric feature set. In this paper, *N* is 12, which is the number of feature sets obtained after the processing described in Section 3.1.

The SPM can be defined as follows [28]:

(8) $$\mathrm{SPM}\left(x^{i},x^{j}\right)=\mathrm{SID}\left(x^{i},x^{j}\right)\times \tan\left(\sqrt{\mathrm{SBD}{\left(x^{i},x^{j}\right)}^{2}+\mathrm{SSD}{\left(x^{i},x^{j}\right)}^{2}}\right)$$

where the spectral brightness difference (SBD) represents the average distance and difference in brightness between spectral vectors [29]. The spectral shape (SSD) is characterized as the difference in spectral shape of two vectors [28]. The spectral information divergence (SID) characterizes the difference in spectral information between different target spectral vectors [28,29]. Smaller SPM values indicate a greater similarity between the given target pixels and the unknown pixels [28]. In this study, SPM is normalized to 0–1 for logical comparison with other spectral matching parameters.

The SBD is characterized by the geometrical distance between two polarimetric feature vectors, which is expressed by the transformation of the Euclidean distance as below [28,29,30]:

(9) $$\mathrm{SBD}\left(x^{i},x^{j}\right)=\sqrt{\frac{1}{N}\times \overset{N}{\underset{k=1}{\sum }}{\left(r_{ik}-r_{jk}\right)}^{2}}$$

Here, *N* denotes the vector dimension used to remove the correlation between the vector size and dimension. Therefore, the SBD represents the average distance between the vectors and ranges from 0–1.

The SSD can be expressed by the transformation of the Pearson correlation coefficient as follows [28,29,30]:

(10) $$\mathrm{SSD}\left(x^{i},x^{j}\right)={\left(\frac{1-\mathrm{SCM}\left(x^{i},x^{j}\right)}{2}\right)}^{2}$$

and

(11) $$\mathrm{SCM}\left(x^{i},x^{j}\right)=\frac{\sum_{k=1}^{N}\left(r_{ik}-\bar{r_{i}}\right)\left(r_{jk}-\bar{r_{j}}\right)}{{\left(\sum_{k=1}^{N}{\left(r_{ik}-\bar{r_{i}}\right)}^{2}\right)}^{1/2}\times {\left(\sum_{k=1}^{N}{\left(r_{jk}-\bar{r_{j}}\right)}^{2}\right)}^{1/2}}$$

where SCM represents the Pearson correlation coefficient with a range from −1 to 1 and the SSD ranges from 0–1. If all the components *x^i^_N_* and *x^j^_N_* are assumed to be non-negative due to the properties of scattering, then the vectors *x^i^* and *x^j^* can be normalized to find the probability vectors *p_ik_* and *p_jk_*. The SID measure is defined using *p_ik_* and *p_jk._* and is given by the following [28,30]:

(12) $$SID\left(x^{i},x^{j}\right)=D\left(x^{i}||x^{j}\right)+D\left(x^{j}||x^{i}\right)=\overset{N}{\underset{k=1}{\sum }}P_{ik}\times \log\left(\frac{P_{ik}}{P_{jk}}\right)+\overset{N}{\underset{k=1}{\sum }}P_{jk}\times \log\left(\frac{P_{jk}}{P_{ik}}\right)$$

where

(13) $$P_{ik}=\frac{r_{ik}}{\sum_{k=1}^{N}r_{ik}};\;P_{ik}=\frac{r_{jk}}{\sum_{k=1}^{N}r_{jk}}$$

SID represents the relative entropy between the vectors using the Kullback-Leibler divergence calculation, and is normalized to 0–1 for logical comparison.

The implementation process of spectral similarity matching model is as follows:

1. Get the average multi-polarimetric features curve of the target sample points (thick oil slick, for example) extracted from the image as the known reference curve, *x^i^* = (*x^i^*_1_, *x^i^*_2_, *x^i^*_3_, …, *x^i^_N_*)^T^
2. Obtain the SPM result of the known reference curve and the whole categorizing images by the pixel-by-pixel similarity calculation.
3. Calculate and obtain the optimal threshold by Otsu image segmentation method to extract the thick oil area with the highest similarity to their spectral vector size, spectral curve shape, and spectral information content.

### 3.3. Comparison of Spectral Similarity Measures

Spectral matching methods are widely used in hyperspectral data processing and matching. Four representative methods—the Euclidian distance (ED), spectral angle measure (SAM), SID measure, and shape measure/spectral correlation similarity (SCS)—are compared with SPM to evaluate its accuracy and demonstrate its effectiveness [27,28,29,30]. The SAM is defined as the inverse cosine of angle between two polarimetric feature vectors with a range from 0 to 1 [27]. If the angle approaches 0°, the similarity between two polarimetric feature vectors increases. If the angle approaches 90°, the similarity between two polarimetric feature vectors decreases. This is defined as follows:

(14) $$\mathrm{SAM}\left(x^{i},x^{j}\right)=\cos^{-1}\left(\frac{\sum_{k=1}^{N}x_{k}^{i}\times x_{k}^{j}}{\sqrt{\sum_{k=1}^{N}{\left(x_{k}^{i}\right)}^{2}\times \sum_{k=1}^{N}{\left(x_{k}^{j}\right)}^{2}}}\right)$$

The ED is used to measure and evaluate the separation or proximity of a given target sample and an unknown sample. The SCS can be used as a similarity measure to calculate the difference in the shape of the curve for two samples. The spectral similarity value, ED, and SCS ranges from 0–1 by normalized, and are defined by the following [28,29,30]:

(15) $$\mathrm{ED}\left(x^{i},x^{j}\right)=\|x^{i},x^{j}\|=\sqrt{\overset{N}{\underset{k=1}{\sum }}{\left(x_{k}^{i}-x_{k}^{j}\right)}^{2}}$$

and

(16) $$\mathrm{SCS}\left(x^{i},x^{j}\right)=\frac{\sum_{k=1}^{N}\left(x_{k}^{i}-\mu^{i}\right)\times \left(x_{k}^{j}-\mu^{j}\right)}{\left(N-1\right)\sigma^{i}\sigma^{j}}$$

where *μ_i_* and *μ_j_* represent the means of a given target sample *x_i_* and an unknown sample *x_j_*, respectively; and *σ_i_* and *σ_j_* represent the standard deviation of *x_i_* and *x_j_*, respectively.

## 4. Results

The proposed method was tested on the two cases of SAR images described in Table 1. The classification accuracies of the different measures were evaluated using the producer’s accuracy (PA), user’s accuracy (UA), average accuracy (AA), and Kappa coefficient (Kappa). The AA is based on the average results of PA and UA of all targets (seawater, thick oil slick, and thin oil slick) [26].

**Figure 6:**
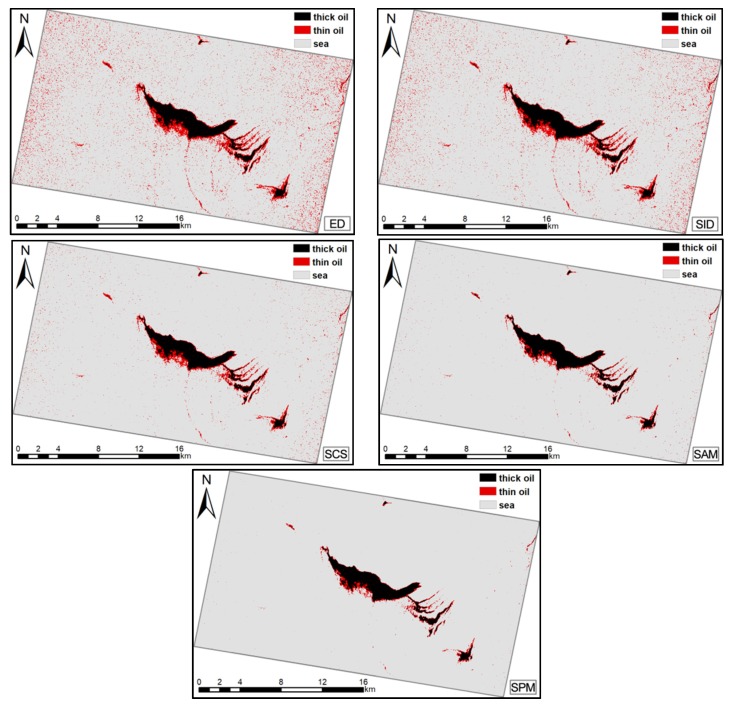
Classification results for Case 1: oil slick maps produced using Euclidian distance (ED), spectral information divergence (SID), spectral correlation similarity (SCS), spectral angle measure (SAM), and spectral pan-similarity measure (SPM).

The results for Case 1 are presented in Table 3 and Figure 6. Compared to the ED, SCS, SID, and SAM results, SPM yields a better AA and Kappa for the study area. The combined UA and PA results for various targets were also better. The SAM achieved results that were comparable to the AA and Kappa for SPM; therefore, the SAM could represent an alternative strategy for the SPM.

Higher PA values represent a lower omission rate. For seawater classifications, SPM had the highest PA (99.95%). However, for thick oil, ED had the highest PA (98.77%); yet, this was only 2.74% greater than that for SPM. For thin oil, SID has the highest PA (66.4%) and it surpassed that for SPM by approximately 20%.

Higher UA values indicate a lower commission rate. As described above, the large seawater sample meant that the UA results for seawater were comparable for each measurement. For seawater, the highest UA was acquired using the SID; however, this was only 0.34% higher than that for the SPM. For thick oil, the best UA was obtained using the SCS (92.41%), which was only 0.58% higher than for the SPM. For thin oil, the SPM achieved the highest UA (75.53%) and surpassed those for the ED (50.88%), SCS (32.94%), SID (57.73%), and SAM (13.42%). The classification results map also shows the advantages of the SPM. The ED and SID results show that seawater was frequently misclassified as an oil slick; this was less of a problem for the SCS and SAM, but the SPM produced the best classification results. The comprehensive accuracy results show that the SPM performed better than the other algorithms.

**Figure 7:**
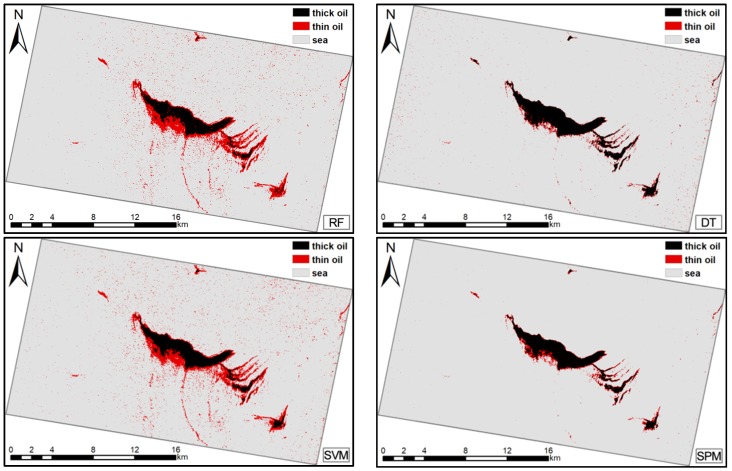
Classification results for Case 1: oil slick maps produced using the random forest (RF), decision tree (DT), support vector machine (SVM), and spectral pan-similarity measure (SPM).

**Figure 8:**
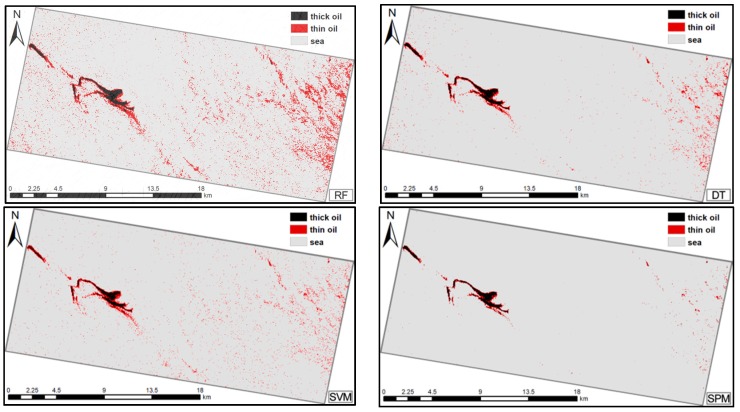
Classification results for Case 2: oil slick maps produced using the random forest (RF), decision tree (DT), support vector machine (SVM), and spectral pan-similarity measure (SPM).

Support vector machine (SVM), decision tree (DT), and Random Forest (RF), the classical machine learning techniques for target extraction and classification [44,62,63,64,65], were used to further demonstrate the advantages and robustness of the proposed method. To provide the same calculation conditions, the same training samples were used as inputs. Quantitative and visual comparisons of the four methods for the two cases are reported in Table 4; Table 5 and Figure 7; Figure 8.

The results show that, for both cases, the AA and Kappa of the proposed method were slightly higher than those of the other three classifiers, except for the average accuracy in Case 2 (difference was no more than 1.5%). In Case 1, for thick oil, the DT produced the highest PA; however, this was only 3.9% higher than the PA of the SPM. Furthermore, the RF produced the highest UA for thick oil, surpassing the SPM by approximately 3.9%. For thin oil, the highest PA was obtained by the RF, and the highest UA was acquired by the SPM. For seawater, the highest PA was obtained by the SPM, and the highest UA was obtained by the SVM, which was slightly higher than that for the SPM. In Case 2, for thick oil, the RF produced the highest PA. For thin oil and seawater, the RF and SPM produced the highest PAs. For thick oil, the SPM obtained the highest UAs. For thin oil and seawater, the SPM and SVM produced the highest UAs. Overall, all four methods produced reasonable classification results, but the SPM was slightly more comprehensive than the other three classifiers. This demonstrates the effectiveness and potential of the proposed method.

## 5. Discussion

### 5.1. Analysis of the Oil Spill Detection Ability of the Proposed Method

The proposed method consists of two parts: the multi-polarimetric features model and the SPM matching algorithm. First, the J–M distance is utilized to evaluate the separability of the polarimetric feature parameters of oil and seawater, thick oil and thin oil, and thin oil and look-alikes, and to select the effective polarimetric features set with a high J–M result. The multi-polarimetric features model is generated by the characteristic curve of the multi-polarimetric features for oil slicks and seawater, which can help determine the intrinsic polarimetric features curve pattern for each class. Second, the SPM matching method based on the multi-polarimetric features model is proposed to search for and match the same category objects with similar spectral vectors, curve shapes, and information content.

The advantages of SPM have been demonstrated in terms of both classification accuracy and visual results compared to the other four classical spectral similarity measures because the former comprehensively considers three types of spectral information instead of only a single index. The effectiveness of the proposed method was also demonstrated via comparison with the RF, SVM, and DT. The proposed method achieved the highest AA and Kappa in both cases (except for the AA in Case 2 within the difference of 1.5%). Although its performance varied for different targets, the proposed method achieved the best or comparable classification accuracy results; when there was a difference, it was no more than 4%, which indicates that the proposed method can achieve a satisfactory performance. However, relatively poor results were obtained for thin oil slicks in both cases, as shown in Table 4 and Table 5. The accuracy results for the four methods were lower in Case 2 than in Case 1. This is because the area of the oil slick was smaller in Case 2, resulting in the oil-water mixing being more sufficient; therefore, the differences in the classification results were a result of changes in the characteristics of the oil slick. In addition, RF performed well in some categories in both cases, especially in thin oil. However, RF exhibited over-fitting results, which caused many seawater samples to be misclassified as thin oil. This is due to the fact that RF achieved attractive and better result when multi-dimensional data inputs were used [66]. In this paper, the low dimension features applied in two cases, and the noise and small training samples size may affect the classification accuracy in the Case 2. In summary, the overall performance of the proposed method was still comprehensively better than that of the SVM, RF, and DT.

### 5.2. Computational Complexity Analysis

For dealing with the SAR data with the multi-polarimetric features dimension *d* (In this paper, the dimension *d* is 12), if ignoring the multi-polarimetric feature extraction and modeling (the computational complexity is *O*(*n*^2^)), all the spectral similarity measures are performed by traversing each pixel of the SAR image, and the highest order of computational complexity is square order *O*(*n*^2^). ED, SCS, SAM and SID require square order *O*(d*n^2^) operations. The computational complexity of the proposed method with three parallel computing is square order *O*(3d*n^2^), which is same highest order as those of other spectral similarity methods. The computational complexity of SVM is cubic order *O*(d*n^3^), which depends on the number of support vectors and Gaussian kernel. The cache size can be adjusted to reduce computational complexity from *O*(d*n^3^) to *O*(d*n^2^). The computational complexity of RF is polynomial order *O* (k*d*n^2^*log n), where *k* is the number of decision trees in RF. The DT require linear logarithmic order *O* (d*n*log n) operations. The main disadvantage of the spectral similarity measure is performed by the pixel-by-pixel similarity calculation, and combined three types of spectral information, and integrates the magnitude of the spectral vector, curve shape, and information content; these need a longer operation time than other spectral similarity measures under the same computational complexity operations.

To summarize, SVM has the highest computational complexity, followed by spectral similarity measures (ED, SCS, SAM, SID and SPM) and the lowest by DT (*O* (n*log n) < *O*(*n*^2^) < *O* (k*n^2^*log n) < *O*(*n*^3^)). However, SPM has the better performance in classification accuracy than ED, SCS, SAM, and SID under the same computational complexity operations. In addition, SPM is still better than the RF, SVM, and DT, which is still valuable and acceptable when considering computational complexity and accuracy. Further research on this issue will be implemented to improve the multiple parallel optimization algorithms and reduce the computational complexity.

## 6. Conclusions

We proposed a SPM matching algorithm based on the multi-polarimetric features model method to evaluate the similarity of features curves. First, the J–M distance is used to evaluate the ability of common polarimetric features to distinguish between targets; from this, an effective polarimetric feature set is selected. Second, the SPM is used to search for and match homogenous objects. The advantages of the SPM in multi-polarimetric feature model matching prove its effectiveness for oil spill detection in comparison with other classical spectral measures methods. The effectiveness of the proposed method is also supported by comparison with the RF, SVM, and DT classifiers. Experiments demonstrated that the proposed method has the greatest accuracy. The AA and Kappa of the proposed method were 84.55% and 0.8855, respectively, which is higher 1.5–7.9% and 1–25% higher than other traditional spectral similarity measures. Furthermore, in most cases the proposed method produces results that are better than RF, SVM and DT. Even in some cases, RF and SVM achieve the best results, the differences between the results of the proposed method and the highest accuracies result were less than 4%, however, relatively poor results were obtained for thin oil slicks in both cases. In addition, RF results perform overfitting. In summary, the proposed method achieved better classification result compared to the other traditional spectral similarity measure under the same computational complexity operations. In addition, compared to three classifiers (RF, SVM, and DT), the proposed method produced better results than RF, SVM and DT by comprehensively considering accuracy and computational complexity.

In the future, further research on separability measure in polarimetric features selection will be implemented to compare and analyze with other feature selection methods. In addition, the separability measure method will also be improved based on other automatic screening and sorting functions, such as the built-in mechanism of RF. For model matching, we will look to develop an automatic multi-polarimetric feature matching model using multiple parallel computations, efficient extraction of a region of interest, and optimization algorithms. In addition, the proposed multi-polarimetric features model is generated by the current common polarimetric features that have previously been used in oil spill studies under different SAR sensors, imaging modes, and the conditions [2,11,15,16,41,50,51,52,53,54,55,56,57,58]. Hence, the proposed method can be extended to different SAR sensors, imaging modes, and environmental conditions in theory, but the multi-polarimetric features model and accuracy may vary and be limited by sea conditions, NESZ, incidence angle, and other factors, due to the difference of SAR sensors design and detection conditions. In summary, we have planned experiments, comparisons, and discussion to use the proposed methods on different types of oil, with different sensors and imaging modes even under different environmental conditions (wind speed, sea currents, waves, incidence angle, and NESZ). Moreover, the quantitative effects of different noise levels on the polarization characteristic parameters will also be discussed.

## Figures and Tables

**Table 1:** Properties of the SAR data in two separate cases.

Sensor	RADARSAT-2
Owner/Operator	CSA/MDA
Date	8 May 2010
Time (UTC)	12:01 a.m.
Mode/Product/Polarization	Fine Quad-Pol mode SLC (HH, HV, VH, VV)
Centre Frequency	C-band 5.405 GHz
Slicks present	Natural Crude Oil Seeps
Resolution (Rg × Az)	5.2 × 7.6 (m)
Pixel space (Rg × Az)	4.7 × 5.1 (m)

**Table 2:** Polarimetric features investigated in this study with their expected behavior over the oil slicks and seawater.

Polarimetric Feature	Definition	For Oil	For Sea Surface	References
Alpha (α)	α = P_1_α_1_ + P_2_α_2_ + P_3_α_3_, $P_{i}=\frac{\lambda_{i}}{\sum_{i=1}^{3}\lambda_{i}}$	Higher	Lower	[11,16,41]
Entropy (H)	$H=\sum_{i=1}^{3}-P_{i}\log_{3}P_{i}$	Higher	Lower	[11,16,41,50]
Anisotropy (A)	$A=\frac{\lambda_{2}-\lambda_{3}}{\lambda_{2}+\lambda_{3}}$	Higher	Lower	[11,15,41]
Combination of H and A	(1 − H)*(1 − A)	Lower	Higher	[41]
	(1 − H)*A	Lower	Higher	
	H*(1 − A)	Higher	Lower	
	H*A	Higher	Lower	
Eigenvalues of coherence matrix	λ_1_ ($T_{3}=U_{3}\Sigma U_{3}^{-1}\sum_{i=1}^{i=3}\lambda_{i}u_{i}u_{i}^{T*}$)	Lower	Higher	[11,51,52]
	λ_2_	Lower	Higher	[11,51]
	λ_3_	Lower	Higher	[11,51]
Anisotropy12 (A_12)	$A_{12}=\frac{\lambda_{1}-\lambda_{2}}{\lambda_{1}+\lambda_{2}}$	Lower	Higher	[2,15]
Combination of H and A_12	(1 − H)*(1 − A_12_)	Lower	Higher	[2]
	H*A_12_	Lower	Higher	
	H*(1 − A_12_)	Higher	Lower	
	(1 − H)*A_12_	Lower	Higher	
F	F = [H + A + ρ_CO_ + α]	Higher	Lower	[53]
F_wang	F_wang = [(1 − H) + (1 − α) + A_12_ + ρ_CO_]/4	Lower	Higher	[54]
Surface Scattering Fraction (τ)	$\tau =\frac{\langle \left|S_{HH}+S_{VV}\right|\rangle^{2}}{span}$	Lower	Higher	[43]
Pedestal Height (PH)	$\mathrm{PH}=\frac{\lambda_{3}}{\lambda_{1}}$	Higher	Lower	[41,55,56]
Co-polarization Ratio (PR)	PR= S_VV_^2^/S_HH_^2^	Higher	Lower	[15,38,41,42]
tan(α)	$\tan\left(\alpha \right)\approx \frac{\left|R_{HH}-R_{VV}\right|}{\left|R_{HH}+R_{VV}\right|}$ $R_{HH}=\frac{\cos\emptyset_{i}-\sqrt{\varepsilon_{r}-\sin^{2}\emptyset_{i}}}{\cos\emptyset_{i}+\sqrt{\varepsilon_{r}-\sin^{2}\emptyset_{i}}}$ $\;R_{VV}=\frac{\left(\varepsilon_{r}-1\right)\left(\sin^{2}\emptyset_{i}-\varepsilon_{r}\left(1+\sin^{2}\emptyset_{i}\right)\right)}{\left(\varepsilon_{r}\cos\emptyset_{i}+\sqrt{\varepsilon_{r}-\sin^{2}\emptyset_{i}}\right)}$ *ϕ*_i_: incidence angle *ε*_r_: dielectric constant	Lower	Higher	[11,41]
Cross-polarization ratio (PX)	$\mathrm{PX}=\frac{\langle {\left|s_{HV}\right|}^{2}\rangle }{\langle {\left|s_{HH}\right|}^{2}\rangle +\langle {\left|s_{VV}\right|}^{2}\rangle }$	Higher	Lower	[51]
Polarization Difference (PD)	PD = S_VV_^2^ − S_HH_^2^	Lower	Higher	[51,52,57,58]
The Magnitude of Correlation Coefficient (ρ_co)	$\rho_{\mathrm{co}}=\left|\frac{\langle S_{HH}S_{VV}^{*}\rangle }{\sqrt{\langle {\left|S_{HH}\right|}^{2}\rangle \langle {\left|S_{VV}\right|}^{2}\rangle }}\right|$	Lower	Higher	[15,39,51,52,54]
Polarisation_Fraction (PF)	$\mathrm{PF}=1-\frac{3\lambda_{1}}{\lambda_{1}+\lambda_{2}+\lambda_{3}}$	Lower	Higher	[41,51]

**Table 3:** Classification accuracy assessment with different spectral similarity measures for the data in Case 1.

	**Class**	**Thick Oil**	**Thin Oil**	**Seawater**
**Accuracy**				
ED	PA (%)	98.77	51.43	98.51
	UA (%)	86.76	24.65	99.86
	AA (%)	76.66		
	Kappa	0.7348		
	**Class**	**Thick Oil**	**Thin Oil**	**Seawater**
**Accuracy**				
SCS	PA (%)	95.83	63.68	99.26
	UA (%)	92.41	42.59	99.82
	AA (%)	82.265		
	Kappa	0.8250		
	**Class**	**Thick Oil**	**Thin Oil**	**Seawater**
**Accuracy**				
SID	PA (%)	97.27	66.40	97.10
	UA (%)	90.64	17.80	99.89
	AA (%)	78.18		
	Kappa	0.6304		
	**Class**	**Thick Oil**	**Thin Oil**	**Seawater**
**Accuracy**				
SAM	PA (%)	97.66	49.06	99.76
	UA (%)	89.98	62.11	99.75
	AA (%)	83.05		
	Kappa	0.8737		
	**Class**	**Thick Oil**	**Thin Oil**	**Seawater**
**Accuracy**				
SPM	PA (%)	96.03	44.36	99.95
	UA (%)	91.83	75.53	99.65
	AA (%)	84.55		
	Kappa	0.8855		

**Table 4:** Classification accuracy assessment for the spectral pan-similarity measure (SPM), random forest (RF), support vector machine (SVM), and decision tree (DT) using the data from Case 1.

	**Class**	**Thick Oil**	**Thin Oil**	**Seawater**
**Accuracy**				
SPM	PA (%)	96.03	44.36	99.95
	UA (%)	91.83	75.53	99.65
	AA (%)	84.55		
	Kappa	0.8855		
	**Class**	**Thick Oil**	**Thin Oil**	**Seawater**
**Accuracy**				
RF	PA (%)	90.66	88.43	98.83
	UA (%)	95.78	33.10	99.88
	AA (%)	84.4		
	Kappa	0.807		
	**Class**	**Thick Oil**	**Thin Oil**	**Seawater**
**Accuracy**				
SVM	PA (%)	94.38	83.77	98.45
	UA (%)	95.07	32.52	99.95
	AA (%)	84.02		
	Kappa	0.7601		
	**Class**	**Thick Oil**	**Thin Oil**	**Seawater**
**Accuracy**				
DT	PA (%)	99.98	22.26	99.64
	UA (%)	84.52	33.13	99.87
	AA (%)	73.23		
	Kappa	0.8592		

**Table 5:** Classification accuracy assessment for the spectral pan-similarity measure (SPM), random forest (RF), support vector machine (SVM), and decision tree (DT) using the data from Case 2.

	**Class**	**Thick Oil**	**Thin Oil**	**Seawater**
**Accuracy**				
SPM	PA (%)	79.04	17.67	99.60
	UA (%)	97.70	29.22	98.70
	AA (%)	70.32		
	Kappa	0.6008		
	**Class**	**Thick Oil**	**Thin Oil**	**Seawater**
**Accuracy**				
RF	PA (%)	95.59	61.28	80.51
	UA (%)	72.87	28.02	92.65
	AA (%)	71.81		
	Kappa	0.55		
	**Class**	**Thick Oil**	**Thin Oil**	**Seawater**
**Accuracy**				
SVM	PA (%)	67.14	32.01	98.22
	UA (%)	97.1	18.68	98.91
	AA (%)	68.77		
	Kappa	0.4945		
	**Class**	**Thick Oil**	**Thin Oil**	**Seawater**
**Accuracy**				
DT	PA (%)	82.70	21.80	98.56
	UA (%)	96.46	21.74	98.88
	AA (%)	70.02		
	Kappa	0.5472		
